# A chromosome-level genome assembly of a deep-sea symbiotic Aplacophora mollusc *Chaetoderma* sp.

**DOI:** 10.1038/s41597-024-02940-x

**Published:** 2024-01-25

**Authors:** Yue Wang, Minxiao Wang, Jie Li, Junlong Zhang, Linlin Zhang

**Affiliations:** 1 CAS and Shandong Province Key Laboratory of Experimental Marine Biology, Institute of Oceanology, Chinese Academy of Sciences, Qingdao, 266071 China; 2 Laboratory for Marine Biology and Biotechnology, Qingdao National Laboratory for Marine Science and Technology, Qingdao, China; 3 Center of Deep-Sea Research, Institute of Oceanology, Chinese Academy of Sciences, Qingdao, 266071 China; 4 Key Laboratory of Breeding Biotechnology and Sustainable Aquaculture, Chinese Academy of Sciences, Wuhan, 430072 China; 5 College of Marine Science, University of Chinese Academy of Sciences, Beijing, 100049 China; 6 Department of Marine Organism Taxonomy & Phylogeny, Institute of Oceanology, Chinese Academy of Sciences, Qingdao, 266071 China

**Keywords:** Data publication and archiving, Marine biology

## Abstract

The worm-shaped, shell-less Caudofoveata is one of the least known groups of molluscs. As early-branching molluscs, the lack of high-quality genomes hinders our understanding of their evolution and ecology. Here, we report a high-quality chromosome-scale genome of *Chaetoderma* sp. combining PacBio, Illumina, and high-resolution chromosome conformation capture sequencing. The final assembly has a size of 2.45 Gb, with a scaffold N50 length of 141.46 Mb, and is anchored to 17 chromosomes. Gene annotations showed a high level of accuracy and completeness, with 23,675 predicted protein-coding genes and 94.44% of the metazoan conserved genes by BUSCO assessment. We further present 16S rRNA gene amplicon sequencing of the gut microbiota in *Chaetoderma* sp., which was dominated by the chemoautotrophic bacteria (phylum Gammaproteobacteria). This chromosome-level genome assembly presents the first genome for the Caudofoveata, which constitutes an important resource for studies ranging from molluscan evolution, symposium, to deep-sea adaptation.

## Background & Summary

The Aplacophora is a particular understudied molluscs that is evolutionarily and ecologically important in marine benthic fauna. As early-branching molluscs, Aplacophora is unusual as it is with worm-shaped, shell-less body plan, and covered by cuticle and calcareous sclerites. Two groups Caudofoveata and Solenogastres are often collectively referred to as Aplacophora^1,2^. The pedal groove does not exist in the ventral site of Caudofoveata, which distinguishes it from Solenogastres. Besides, Caudofoveata has gills at the tail of the body and is absence of a foot^1^. Caudofoveata has a worldwide distribution in benthic marine habitats and lives by burrowing in marine soft sediment feeding on organic contents or foraminiferans and small particles^2,3^. Due to the collection difficulty, Caudofoveata is one of the least known classes of Mollusca, with only 142 species (World Register of Marine Species, 2023). To data, a series of studies have researched their taxonomy^4–6^, phylogeny^2,7–11^, ecology^3,12,13^, and evolutions^14–17^.

Caudofoveata had been thought to be the earliest extant offshoots in Mollusca based on its unique body plan and shell-less morphological characters^18,19^. Phylogenomic analyses revealed that Mollusca included two clades, Aculifera (Caudofoveata, Solenogastres and polyplacophorans) and Conchifera (Gastropoda, Bivalvia, Cephalopoda, Scaphopoda and Monoplacophora)^10,11^. The fossil *Kimberella quadrata* was thought to be a stem-group mollusc and had certain traits similar to Aculifera, which indicated Caudofoveata was an early-branching Mollusca^18,20,21^. Overall, Caudofoveata is central to understand the origin and evolutionary history of molluscs, which is the second most diverse metazoan animal group.

Aplacophora is of particular interest from the benthic fauna of the deep sea especially regarding to its diversity and adaptation. With deep sea expeditions increasing in the Atlantic Ocean and the Northwest Pacific, more and more new Aplacophoran molluscs were described and studied^3,22–25^. Almost 86% of the Aplacophora were found at depths of more than 200 m^1^ and some species showed high abundances at deep-sea benthos^26^. *Prochaetoderma yongei*, a widespread deep sea Caudofoveata species in Atlantic, was thought to be successful due to its omnivorous and rapid development ability^3,26^. *Helicoradomenia* spp. which is a Solenogastres species has been found in the sulfide-based chemosynthetic hydrothermal vent with epi- and endocuticular bacterial symbionts^27^. By investigating the food sources and anatomy of 200 individuals within 60 candidate deep-sea Solenogastres species, researchers revealed a high degree of food specialization with modifications in the radula, foregut, and glands morphologies^28^. Considering their great ecological importance and diverse adaptation strategies to deep-sea environment, Aplacophora could be an ideal group to study deep-sea adaptation.

Despite the evolutionary and ecological importance, the studies of Caudofoveata are hampered by the lack of genomic resources. Here, we generated a high-quality chromosome-level genome of *Chaetoderma* sp. for the first time in the Caudofoveata based on PacBio long reads, Illumina short reads, and high-resolution chromosome conformation capture (Hi-C) sequencing reads. The final assembly of *Chaetoderma* sp. was 2.45 Gb, consisting of 17 chromosomes with scaffold N50 length of 141.46 Mb. We predicted 23,675 protein-coding genes from the genome of *Chaetoderma* sp. by integrating de-novo, homologous, and transcriptome annotation methods as well as manual correction. Through the analysis of intestinal microbial composition of *Chaetoderma* sp., we discovered that SUP05, a group of chemoautotrophic bacteria was the dominant bacterial community in the gut, indicating a potential symbiotic relationship between them. The resulting genome assembly, annotation, and report of symbiotic bacteria by 16S rRNA gene amplicon sequencing will provide a valuable resource for further studies of the Caudofoveata and for molluscan evolution and deep-sea ecology in general.

## Methods

### Sample collection and sequencing

The *Chaetoderma* sp. specimens were collected from Site F methane seep^29^ (also known as Formosa Ridge) by the TV grab in the South China Sea during the voyage of the scientific research ship KEXUE from 2020 to 2022. When the samples were collected by the TV grab onto the ship, they were flash-frozen in liquid nitrogen immediately and stored in −80 °C refrigerator. The same frozen specimen of *Chaetoderma* sp. was used to perform the Illumina, PacBio and Hi-C sequencing. The total genomic DNA was extracted from the body wall by SDS method and followed by chloroform purification, examination of the quantity and quality through Qubit and Agilent bioanalyzer instrument. The qualified genomic DNA was used to construct libraries.

Firstly, in order to estimate genome complexity, we used physical breaking method to break up the genome DNA to 350 bp fragment, and then built the small fragment sequencing library which was applied to an Novaseq 6000 platform (Illumina, Inc., San Diego, CA, USA). A total of 57.80 Gb 150 bp paired-end sequencing reads were obtained (Table 1). Secondly, the PacBio library was constructed by following the standard protocol of manufacturer (Pacific Biosciences, Menlo Park, CA, USA), including using g-TUBE to break up DNA, the repair of DNA, the connection of dumbbell connector, the digestion of exonuclease and the filtration of target DNA fragment by BluePippin. A total of three SMART cells and 62.4 Gb clean long HiFi reads with 26.01X coverage were sequenced through circular consensus sequencing (CCS) model (Table 1). Thirdly, we applied high-throughput chromatin conformation capture (Hi-C) method to generate a chromosome-level genome. As for Hi-C library, formaldehyde was used to fix cells, and DpnII restriction endonuclease was used to digest cells. Using the terminal repair mechanism, DNA was labelled and cycled. The Hi-C library was built by using streptavidin magnetic beads to selectively capture DNA fragments containing interaction relationships and was evaluated for quality through Qubit 2.0, Agilent 2100 systems and Q-PCR method. Illumina NovaSeq 6000 platform (Illumina, USA) was performed to execute Hi-C sequencing. We obtained 257.18 Gb clean reads in total (Table 1).

**Table 1:** Statistics of sequencing data.

Sequencing technology	Survey	PacBio	Hi-C	16 S Sample1	16S Sample2	16S Sample3
Clean data	57.80 Gb	62.44 Gb	257.18 Gb	28.5 Mb	26.9 Mb	26.9 Mb
Depth (×)	23.30	26.01	104.97	—	—	—
GC content (%)	40.56	—	41.00	48.12	49.30	48.49
Q30 (%)	92.88	—	96.59	92.34	92.55	92.19

To better annotate the genome assembly, we performed transcriptome sequencing of *Chaetoderma* sp. using the frozen body. The total RNA was extracted using Trizol (Invitrogen). Qubit and agarose gel electrophoresis were further applied to examine the concentration and quality of RNA. VAHTS® Universal V8 RNA-seq Library Prep Kit for Illumina (Vazyme #NR605) was used to constructed RNA-seq library. The sequencing library was further sequenced on Novaseq 6000 platform in 150 bp paired-end mode (Illumina, Inc., San Diego, CA, USA). A total of 6.5 Gb raw reads were obtained.

### Genome assembly and Hi-C scaffolding

**Fig. 1:**
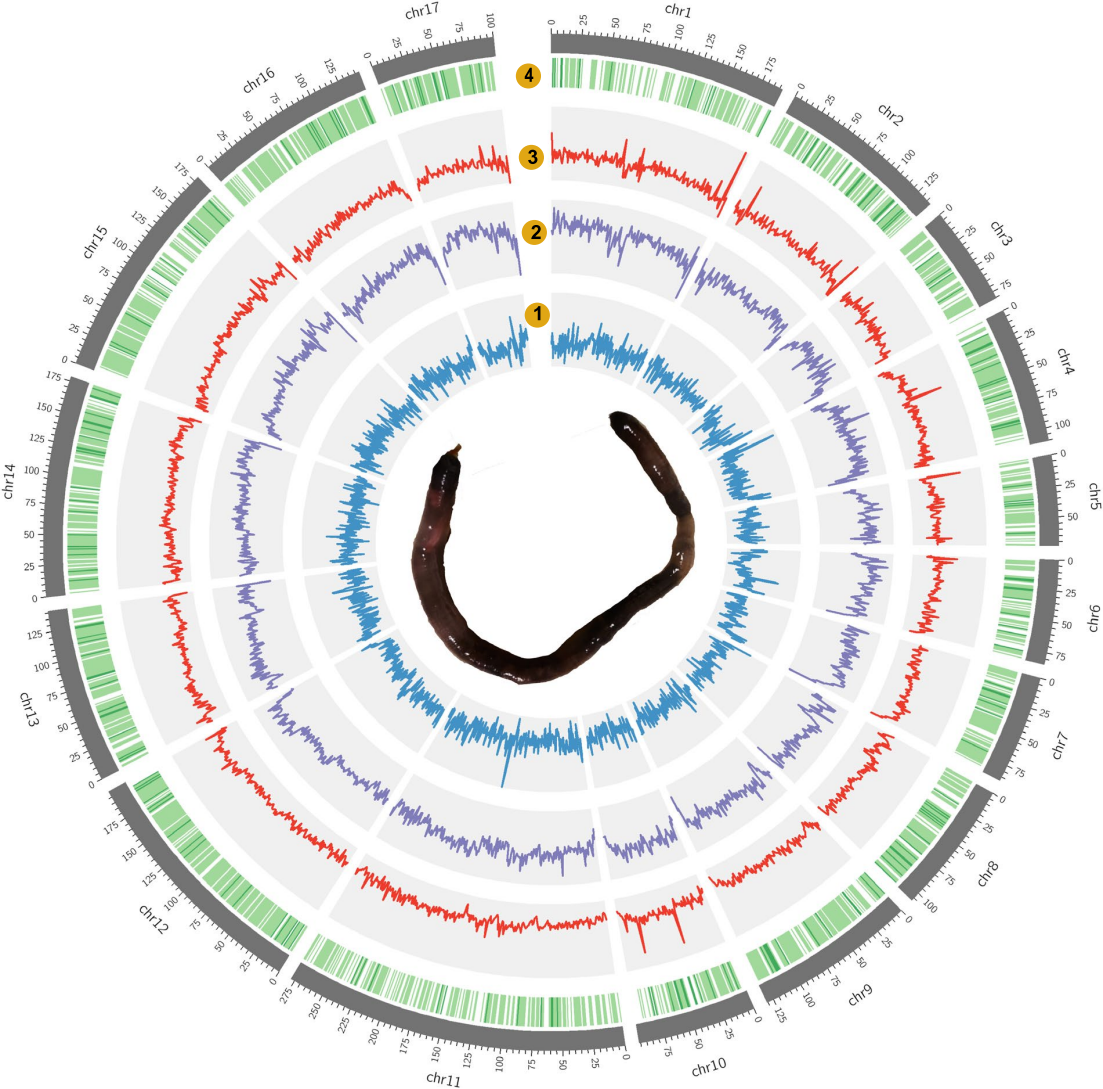
Characterization of the assembled *Chaetoderma* sp. genome. From inner to outer layers: photograph of *Chaetoderma* sp.; distribution of GC content in the genome; repeat sequence density across the genome; LTR density across the genome; gene density across the genome. All the tracks are drawn in 1 Mb sliding windows and chromosome-level scaffolds at scale by circos v0.69-9^65^.

**Fig. 2:**
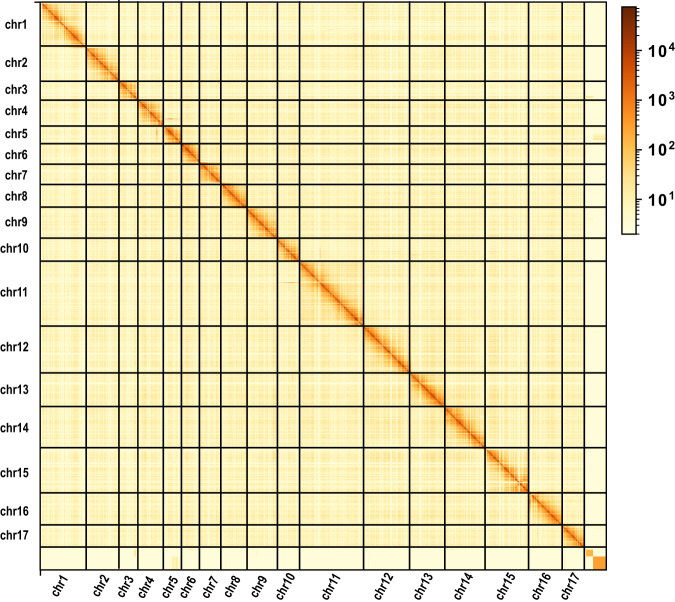
Hi-C chromosome interaction heatmap. Abscissa and ordinate represent order of each bin on corresponding chromosome group. Scale bar illuminates intensity of interaction from white (low) to red (high).

**Fig. 3:**
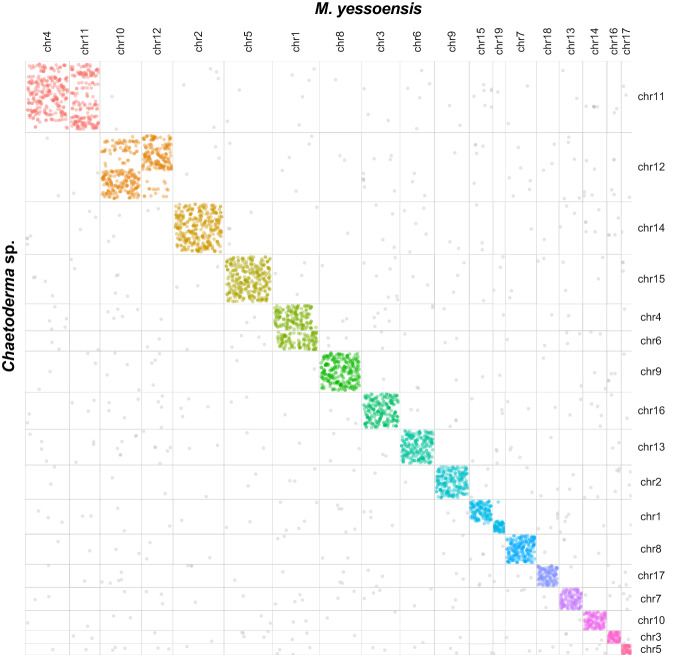
Genomic collinearity between the molluscs *Chaetoderma* sp. and *Mizuhopecten yessoensis*.

Genome size, repetitive sequence ratio, and heterozygosity were first estimated based on Illumina short-read data. We used jellyfish v2.3.0^30^ and GenomeScope v1.0^31^ to analyse the K-mer frequency (k = 21). Based on Illumina reads, the K-mer analysis showed that the genome size of *Chaetoderma* sp. was 2.2 Gb and the heterozygosity was 1.39%. HiFi-asm v0.16^32^ was applied to assemble the genome based on PacBio long-read data. Pilon v1.23^33^ was then used to polish the assembly with the Illumina short-read data. Purge_dups v1.2.5^34^ was used to remove duplication. The assembly of *Chaetoderma* sp. genome is 2.45 Gb, consisting of 5,603 contigs with contig N50 of 1.06 Mb (Table 2). The BUSCO assessment value is 92.03% (metazoan_odb10) and the GC content of the genome assembly is 40.93%. As for Hi-C scaffolding, at first Juicer v1.6^35^ was used to deal with the Hi-C sequencing data and obtain the input file for the next analysis. Then, 3D-DNA v201008^36^ as the core software was used to scaffold the contigs under default settings. Juicebox v1.11.08^35^ was used to visualize chromosome assembly results, choosing the best result from 3D-DNA output, and marking the correct boundary of the chromosome according to the interaction heatmap. Finally, we reran 3D-DNA using corrected assembly result and exported the final chromosome assembly genome. After Hi-C scaffolding, 94.83% genome reads were anchored to 17 chromosomes with scaffold N50 length of 141.46 Mb and BUSCO assessment value of 89.52% (Fig. 1, Tables 2, 4, and 6). The 17 chromosomes were exhibited clearly in the interaction heatmap (Fig. 2) and also had a conserved collinearity relationship with the chromosomes of *Mizuhopecten yessoensis* (Fig. 3). All the bioinformatic software mentioned in this section were used with default parameters.

**Table 2:** Statistics of genome assembly.

Category	Contig (Before Hi-C)	Chromosome
Genome size (Gb)	2.45	2.45
sequence_count	5,603	5,340
N50	1,055,820	141,459,992
N90	165,564	80,506,680
Max Length	6,942,394	277,918,263
Mean Length	196,834	458,691
GC content (%)	40.93	40.93
Illumina mapped rates (%)	95.00

**Table 3:** Statistics of genome annotation.

Category	Number
Protein-coding genes	23,675
rRNA	118
tRNA	4,633
SwissProt	23,492 (99.23%)
Pfam	16,278 (68.76%)
GO	12,019 (50.77%)
KEGG	12,165 (51.38%)

**Table 4:** Statistics of BUSCO assessment after Hi-C.

	Assembly	Annotation
Complete BUSCOs (C)	854 (89.52%)	901 (94.44%)
Complete and single-copy BUSCOs (S)	844 (88.47%)	876 (91.82%)
Complete and duplicated BUSCOs (D)	10 (1.05%)	25 (2.62%)
Fragmented BUSCOs (F)	42 (4.40%)	33 (3.46%)
Missing BUSCOs (M)	58 (6.08%)	20 (2.10%)
Total lineage BUSCOs	954	954

**Table 5:** Statistics of Repeats.

Type	Number	Length	Rate (%)
ClassI:Retroelement	2,261,427	939,883,376	38.43
ClassI/LINE	459,481	202,881,508	8.29
ClassI/SINE	43,451	4,822,975	0.20
ClassI/LTR/Copia	636	540,596	0.02
ClassI/LTR/ERV	31,357	2,820,932	0.12
ClassI/LTR/Gypsy	206,810	165,811,573	6.78
ClassI/LTR/Ngaro	18,258	2,481,937	0.10
ClassI/LTR/Pao	8,223	6,735,094	0.28
ClassI/LTR/Other	1,428,775	454,783,456	18.59
ClassI/DIRS	64,436	99,005,305	4.05
ClassII:DNA transposon	1,742,149	425,182,843	17.38
ClassII/Academ	18,303	13,724,742	0.56
ClassII/CACTA	26,659	3,182,184	0.13
ClassII/Helitron	25,710	8,233,109	0.34
ClassII/Maverick	17,783	12,435,824	0.51
ClassII/Other	15,696	1,834,648	0.08
Unknown	267	62,068	0.00
Total TEs	4,003,846	1,365,129,223	55.81

**Table 6:** Statistics of 17 chromosomes.

Chromosome ID	Length (bp)	Percentage (%)
1	192,189,196	7.85
2	138,656,860	5.66
3	80,506,680	3.29
4	110,372,735	4.51
5	72,989,270	2.98
6	84,401,428	3.45
7	88,103,728	3.60
8	107,653,829	4.40
9	130,592,541	5.33
10	95,945,391	3.92
11	277,9182,63	11.35
12	199,972,391	8.17
13	141,459,992	5.78
14	178,501,283	7.29
15	179,245,392	7.32
16	143,251,023	5.85
17	100,061,905	4.09
Total	2,321,821,907	94.83
Unplaced	126,566,857	5.17

### Annotation of Repetitive Elements

*De novo* repeat library prediction and homology comparison were applied for repeats annotation. We employed RepeatModeler2 v2.0.1^37^ with default parameters to construct the *de novo* repeat library. LTR_FINDER v1.07^38^ and LTR_retriever v2.9.0^39^ were used to identify long terminal repeat (LTR) sequence in the genome by using default parameters. The *de novo* repeat library and LTR library were combined and removed redundancy to generate the final repeat library. RepeatMasker v4.1^40^ (-frag 100000 -gc 33.37 -lcambig -xsmall -gff) was applied to identify repeats with RepBase and *de novo* species-specific library in the genome of *Chaetoderma* sp. The proportion of Transposon elements (TEs) in *Chaetoderma* sp. genome is 55.81%. Among them, retroelement accounts for 38.43%, DNA transposon accounts for 17.38%. The most abundant transposon type is the LTR (Table 5).

### Gene Prediction

The Caudofoveata gene prediction is challenging because of the high ratios of TEs and long introns, as gene prediction programmes may split a single gene into truncated partial-gene models. We employed three different approaches to predict protein-coding genes, homolog-based, transcriptome-based annotation, and *ab initio* gene prediction. Homolog-based annotation was performed by TBLASTN v2.13.0^41^ (-evalue 1e-10) based on homology sequences from *Acanthopleura granulate*, *Crassostrea gigas*. Genewise v2.4.1^42^ (-nosplice_gtag -pretty -pseudo -gff -cdna -trans) was used to predict genes based on homologous proteins. Second, we fully utilized and integrated transcriptome evidence in the gene prediction process, since this evidence can be helpful in the case of high ratios of TEs and long introns. Trinity v2.13.2^43^ was used for transcriptomic level *de novo* assembly with default parameters. Hisat2 v2.2.1^44^ (--skip 8 --qc-filter) was used to align transcriptome data to the genome, StringTie v2.2.1^45^ was used to predict the structure of all transcribed reads. Subsequently, Program to Assemble Spliced Alignment (PASA) v2.5.2^46^ was employed to integrate genome and transcriptome results. Third, *ab initio* gene prediction was carried out on the repeat-masked genome assembly by Braker2 v2.1.6^47^ and Augustus v3.5.0^48^ with default parameters. Finally, EvidenceMolder v1.1.1^49^ was employed to integrate gene models from different prediction tools. We further used tRNAscan-SE v1.3.1^50^ and barrnap v0.9 (http://lup.lub.lu.se/student-papers/record/8914064) to identify tRNA and rRNA by using default parameters. Finally, we predicted 23,675 protein-coding genes from the chromosome-level genome of *Chaetoderma* sp. by integrating *de novo*, homologous, and transcriptome annotation methods as well as modification of several genes’ structure such as Hox by comparing with homolog species one by one manually (Table 3). We used the BUSCO v5^51^ to evaluate the quality of annotation results. The BUSCO completeness score is 94.44%, and the single copy score is 91.82% (Table 4). 23,503 (99.27%) of the protein-coding genes we predicted were annotated through blasting against SwissProt^52^ and interproscan^53^ against pfam^54^ database (Table 3).

### 16S rRNA sequencing and analysis

**Fig. 4:**
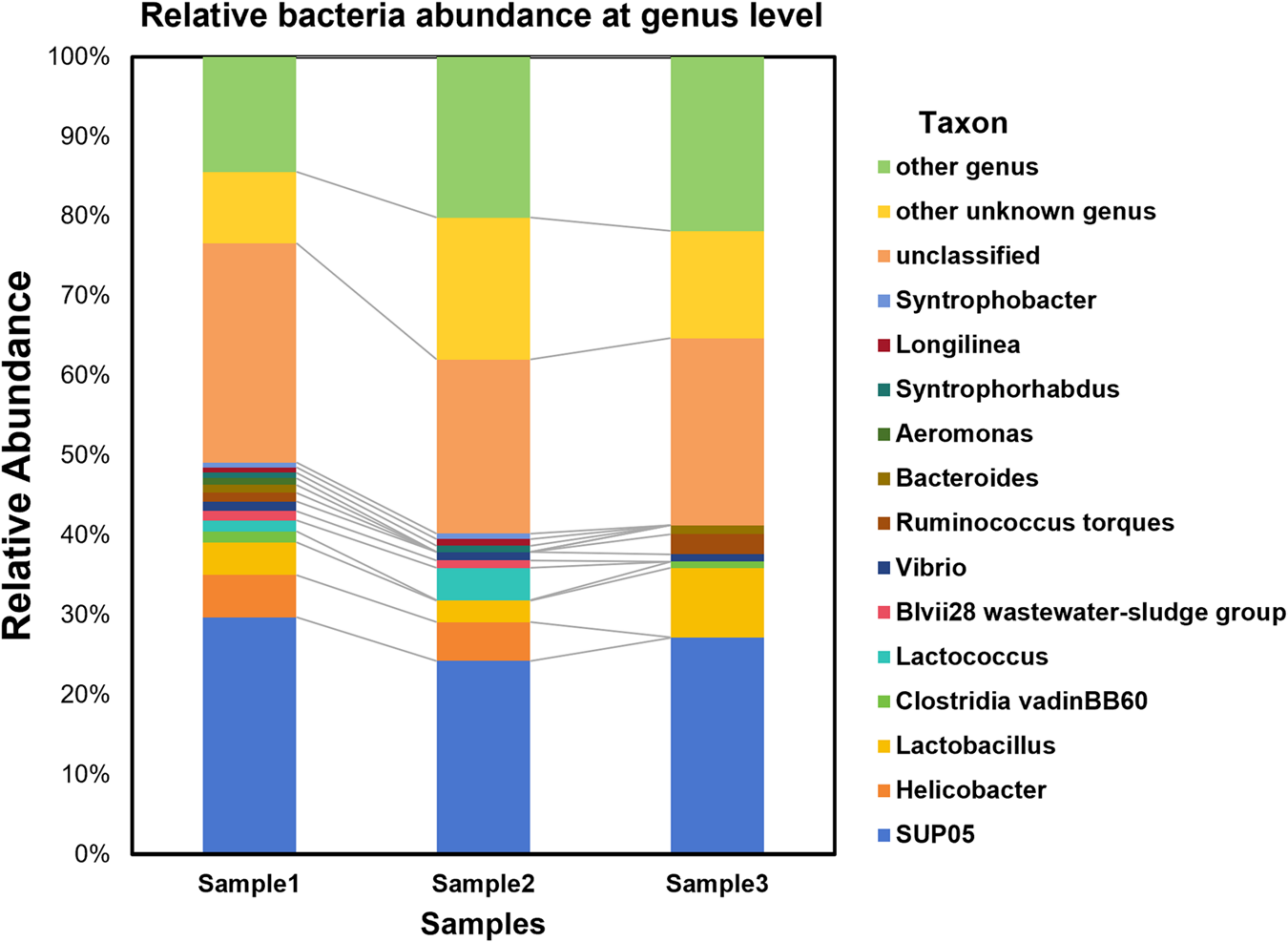
The intestinal microbial composition of *Chaetoderma* sp. SUP05, a group of Gammaproteobacteria with chemoautotrophic ability, was the most abundant bacteria in the intestinal contents of *Chaetoderma* sp.

The total genome DNA of the intestinal contents of *Chaetoderma* sp. was extracted through SDS method. After monitoring the DNA concentration and purity based on 1% agarose gels, DNA was diluted to 1 ng/µL using sterile water. Specific primer (V4: 515F-806R) and barcodes were applied to amplify 16S rRNA genes. The library was sequenced on the Illumina NovaSeq platform to obtain 250 bp paired-end reads. We used FLASH v1.2.11^55^ to merge paired-end reads and used fastp v0.20.0^56^ for data quality control with default parameters. QIIME2 v202006^57^ with default parameters was used to obtain ASVs (Amplicon Sequence Variants) and annotate species based on Silva Database. The result of 16S rRNA sequencing showed that SUP05 was the most abundant bacteria in the intestinal contents of *Chaetoderma* sp. and SUP05 is a group of Gammaproteobacteria with chemoautotrophic ability^58^ (Fig. 4).

## Data Records

The raw Illumina, PacBio, and Hi-C sequencing data are deposited in the NCBI under the accession number SRP457225^59^. The assembled genome sequence is deposited into NCBI under accession number GCA_034401795.1^60^. The genome annotation file is available from the Figshare repository^61^. The SRA database of transcriptome data is SRR26949954^62^. The SRA database of raw Illumina 16S rRNA sequencing is under the accession number SRP458647^63^.

## Technical Validation

### Evaluating genome assembly and annotation completeness

The final assembly of *Chaetoderma* sp.’s genome is 2.45 Gb, consisting of 17 chromosomes with contig N50 of 1.06 Mb and scaffold N50 of 141.46 Mb (Fig. 1, Table 2). The genome size is similar with the result that was estimated by jellyfish. In order to evaluate the genome assembly and annotation, we adopted two methods including Illumina reads remapping using Bowtie2 v2.4.5^64^ and BUSCO v5^51^ assessment using database metazoan_odb10. The alignment rate of Illumina reads was 95% (Table 2). 854 (89.52%) of 954 BUSCOs were included in the assembly of *Chaetoderma* sp. and 901 (94.44%) of 954 BUSCOs were included in the gene models of *Chaetoderma* sp. (Table 4). We also compared our results with other molluscs’ assembly and annotation (Table 7). Overall, these data indicate the genome assembly and annotation of *Chaetoderma* sp. is complete and high-quality.

**Table 7:** The comparison of genome assembly and annotation between *Chaetoderma* sp. and other Molluscs.

Species	Genome size	Contig N50	Scaffold N50	Gene Num	Busco (genome)	Busco (protein)
*Chaetoderma* sp.	2.45 Gb	1.06 Mb	141 Mb	23,675	89.5	94.4
*Acanthopleura granulata*	606.9 Mb	—	24 Mb	25,359	97.4	97.0
*Hanleya hanleyi*	2.52 Gb	—	65.0 kb	34,223 (supported)	79.4	80.9
*Crassostrea gigas*	586.9 Mb	3.1 Mb	61 Mb	30,342	92.1	98.2
*Mizuhopecten yessoensis*	988 Mb	38 kb	804 kb	26,415	94.1	98.7
*Pinctada fucata*	930 Mb	2.56 Mb	64.5 Mb	32,938	95.6	94.6
*Lottia gigantea*	360 Mb	96 kb	1.9 Mb	23,818	95.8	96.5
*Aplysia californica*	927 Mb	9.6 kb	917.5 kb	19,944	92.3	98.6
*Octopus bimaculoides*	2.3 Gb	5.5 kb	96.9 Mb	33,524	90.0	90.2
*Nautilus pompilius*	730 Mb	1.1 Mb	—	17,710	91.3	93.5

## Data Availability

No custom script was used in this work. Software that was used to analyse data was listed in methods in detail and commands were used based on the manuals.
